# Lack of clinically evident signs of organ failure affects ED treatment of patients with severe sepsis

**DOI:** 10.1186/1865-1380-6-4

**Published:** 2013-02-27

**Authors:** Dirkjan Kakebeeke, Alice Vis, Ernie RJT de Deckere, Maro H Sandel, Bas de Groot

**Affiliations:** 1 Leiden University Medical Centre, Leiden, The Netherlands; 2 Medical Centre Haaglanden, The Hague, The Netherlands; 3 HAGA Hospital, The Hague, The Netherlands

**Keywords:** Severe sepsis, Septic shock, Emergency services hospital, “Surviving Sepsis Campaign,” Compliance, Protocol adherence

## Abstract

**Background:**

It is not known whether lack of recognition of organ failure explains the low compliance with the “Surviving Sepsis Campaign” (SSC) guidelines. We evaluated whether compliance was higher in emergency department (ED) sepsis patients with clinically recognizable signs of organ failure compared to patients with only laboratory signs of organ failure.

**Methods:**

Three hundred twenty-three ED patients with severe sepsis and septic shock were prospectively included. Multivariable binary logistic regression was used to assess if clinical and biochemical signs of organ failure were associated with compliance to a SSC-based resuscitation bundle. In addition, two-way analysis of variance was used to investigate the relation between the predisposition, infection, response and organ failure (PIRO) score (3 groups: 1–7, 8–14, 15–24) as a measure of illness severity and time to antibiotics with disposition to ward or ICU as effect modifier.

**Results:**

One hundred twenty-five of 323 included sepsis patients with new-onset organ failure were admitted to the ICU, and in all these patients the SSC resuscitation bundle was started. Respiratory difficulty, hypotension and altered mental status as clinically recognizable signs of organ failure were independent predictors of 100% compliance and not illness severity *per se*. Corrected ORs (95% CI) were 3.38 (1.08–10.64), 2.37 (1.07–5.23) and 4.18 (1.92–9.09), respectively. Septic ED patients with clinically evident organ failure were more often admitted to the ICU compared to a ward (125 ICU admissions, *P* < 0.05), which was associated with shorter time to antibiotics [ward: 127 (113–141) min; ICU 94 (80–108) min (*P* = 0.005)].

**Conclusions:**

The presence of clinically evident compared to biochemical signs of organ failure was associated with increased compliance with a SSC-based resuscitation bundle and admission to the ICU, suggesting that recognition of severe sepsis is an important barrier for successful implementation of quality improvement programs for septic patients. In septic ED patients admitted to the ICU, the time to antibiotics was shorter compared to patients admitted to a normal ward.

## Background

Recent studies showed that increasing compliance with the “Surviving Sepsis Campaign” (SSC) guidelines is associated with reduced mortality in patients with severe sepsis and septic shock admitted to the ICU [1-4]. Unfortunately, the same studies also report that compliance is low, ranging from 10 to 52% [1-4]. Besides development of new treatment modalities for sepsis, it is equally important to find reasons for non-compliance and optimize implementation of treatment strategies proven to be beneficial. Causes of non-adherence range from physician-based factors, i.e., lack of awareness, to logistic factors, i.e., lack of staffing [5]. Insight in these factors is crucial for successful guideline implementation [5], because each barrier requires a specific solution. For example, a Dutch tailored intervention project failed to reduce time to antibiotics when targeting logistic barriers in the ED, and administration of antibiotics in the ED instead of the ward reduced time to antibiotics only slightly [6]. In this intervention project, recognition of sepsis was not considered as a possible delaying factor, while this might have been the “bottleneck” and explain why the other measures did not reduce time to antibiotics. Several other studies have investigated behavioral, logistic and economic factors associated with non-adherence [7-9]. Only one retrospective study suggested that lack of recognition of sepsis plays a role in compliance to an SSC-based quality improvement program [10], while recognition of severe sepsis is the first step in optimizing ED sepsis treatment. In addition, not all patients with severe sepsis are admitted to the ICU, i.e. because of age, erroneous clinical judgment or contraindications for ICU admission. Instead, approximately two-thirds of ED patients with severe sepsis are admitted to a normal ward instead of the intensive care unit (ICU), with clinically evident signs of organ failure less frequently present in the ward admissions [11]. Especially for these patients, recognition of organ failure and optimization of ED treatment are important because not all SSC targets can be attained and optimal ED treatment might prevent progression to more severe stages of sepsis and decrease hospital lengths of stay. Once admitted to a normal ward (and not the ICU), patient monitoring is minimal so that clinical deterioration is easily missed and a potential window of opportunity is lost [11-14].

In the present study, it is hypothesized that septic ED patients with clinically recognizable signs of organ failure, i.e., hypotension, are treated better than patients with only biochemical signs of organ failure, i.e., hyperlactatemia. If recognition of organ failure is the “bottleneck” in compliance to the SSC compared to other aforementioned factors, quality improvement programs should develop specific tools for recognition of biochemical signs of organ failure, because both have similar mortality [15]. The advice to screen for signs of organ failure besides screening for the presence of infection and systemic inflammatory response criteria^1^ might be insufficient.

The purpose of this study was therefore to assess if compliance is higher in ED sepsis patients with clinically evident signs of organ failure compared to patients with only biochemical signs of organ failure relative to other potential factors that might affect protocol adherence.

## Methods

### Study design and setting

This was a prospective observational cohort study in the EDs of the Medical Centre Haaglanden (MCH, Westeinde), an urban hospital with ~49,000 patients annually, and the Leiden University Medical Centre (LUMC), a tertiary care university hospital with ~26,000 visits per year. Patients were enrolled between 1 November 2007 to 1 March 2011 in MCH and from 1 May 2009 to 1 March 2011 in LUMC. The SSC-based guidelines were implemented later in the LUMC, resulting in a shorter period of inclusion in the academic center. The study was part of a quality improvement program with the aim to implement SSC-based guidelines [1] and was approved by the medical ethical committee of the MCH. Medical personnel were informed about the study by means of presentations and flyers containing the inclusion criteria and goals to be achieved. In both EDs, one dedicated doctor informed all new nurses and doctors about the campaign. Medical personnel were motivated to attain all ED goals and to consult the ICU if inclusion criteria were met. In the Netherlands, the ED is only managed by qualified ED physicians from 8.00 to 23.00 h during weekdays. During night and weekend shifts, residents of medicine or surgery provide patient care. The ICU physician decided if a patient needed ICU admission.

### Selection of participants

All patients with a suspected infection were screened for systemic inflammatory response syndrome (SIRS) and new onset organ failure criteria according to the SSC [1]. Consecutive ED patients, 18 years and older, meeting the criteria for severe sepsis and septic shock were included.

### Methods of measurement

#### Measurements

Compliance to the SSC resuscitation bundle was quantified by assigning one point to each of the following goals attained: lactate measurement within 6 h, blood cultures before antibiotics, administration of antibiotics within 3 h, mean arterial pressure above 65 mmHg within 6 h, 1.5 l fluid bolus in case of hypotension below 90 mmHg or lactate >4 mmol/l, and ICU consultation to enable completion of the resuscitation bundle of the SSC. In the Netherlands, treatment requiring central venous and arterial catheters is usually performed in the ICU and not in the ED. The Mortality in Emergency Department Sepsis (MEDS) and predisposition, infection, response, and organ failure (PIRO) scores were used to quantify illness severity as described previously [16,17]. Signs of organ failure as described in the PIRO score were used because these were shown to be associated with mortality.

Screening for suspected infection, SIRS criteria and signs of organ failure was done on standard data collection forms. For included patients, doctors had to fill out goals of the resuscitation bundle, time when goal was initiated and admitting department. Demographic, clinical and laboratory data were recorded prospectively in a digital hospital information system [Table 1, Chipsoft Ezis (Amsterdam, The Netherlands), in the Medical Centre Haaglanden, E-Care (Turnhout, Belgium) in Leiden University Medical Centre]. Time zero was defined as the time of registration. Time of registration, start of antibiotics and fluids, and amount of fluids were also recorded in the hospital information systems. Time to antibiotics and availability of blood results were calculated by subtraction of the time of antibiotics administration/availability, respectively, from the time of registration.

**Table 1:** Patient characteristics

	**Total**	**Ward**	**ICU**	***P*-value**
No. (%)	323	198 (61)	125 (39)	
Age (years)	66 ± 17	68 ± 17	63 ± 16	0.015
Male sex	183 (57)	107	76	0.294
**Comorbidities (%)**				
COPD	49 (15)	32 (16)	17 (14)	0.419
Liver disease	34 (11)	20 (10)	14 (11)	0.850
Malignancy, not metastasized	39 (12)	21 (11)	17 (14)	0.372
Malignancy, metastasized	31 (22)	29 (15)	2 (2)	0.004
Immune compromised†	96 (30)	65 (33)	31 (25)	0.16
**Clinical presentation**				
Respiratory rate (/min)	28 ± 10	27 ± 9	29 ± 10	0.403
SO_2_	92±9	93 ± 7	90 ± 11	0.035
Heart rate (/min)	111 ± 24	110 ± 24	111 ± 25	0.820
Systolic BP (mmHg)	105 ± 31	105 ± 30	106 ± 33	0.763
Diastolic (mmHg)	58 ± 18	57 ± 18	59 ± 18	0.455
Altered mental status	118 (37)	59 (30)	59 (47)	0.005
Febrile chills	72 (22)	49 (25)	23 (18)	0.320
Temperature (°C)	38.1 ± 1.6	38.0 ± 1.4	38.2 ± 1.9	0.354
**Laboratory results**				
Leucocyte count (10 × 9/l)	14.1 ± 9.9	15.1 ± 9.0	12.9 ± 11.2	0.048
Platelets (.10^12^/l)	248 ± 160	258 ± 149	235 ± 178	0.223
INR	1.8 ± 1.5	1.7 ± 1.5	1.9 ± 1.5	0.598
Bilirubin (μmol/l)	22 ± 29	23 ± 33	21 ± 24	0.557
Lactate (mmol/l)	3.7 ± 2.6	3.5 ± 2.5	3.9 ± 2.9	0.289
C-reactive protein (mg/l)	194 ± 142	183 ± 135	216 ± 151	0.046
Glucose (mmol/l)	9.2 ± 5.7	9.4 ± 6.3	8.6 ± 4.0	0.203
pH	7.35 ± 0.59	7.32 ± 0.79	7.39 ± 0.10	0.26
Creatinine (μmol/l)	171 ± 116	175 ± 123	166 ± 106	0.516
Urea (mmol/l)	14.1 ± 10.3	15 ± 11	13 ± 9	0.150
**Suspected site of infection (%)**				
Pneumonia	165 (51)	100 (51)	60 (48)	1.0
Urinary tract infection	97 (30)	66 (33)	31 (25)	0.164
Abdominal	47 (15)	26 (13)	21 (17)	0.259
Neurological	8 (3)	4 (2)	4 (3)	0.479
Skin	22 (7)	8 (4)	13 (3)	0.02
Other	41 (13)	26 (13)	15 (12)	0.862
**Illness severity**				
Number of acute organ dysfunctions	1.8 ± 0.9	1.7 ± 0.9	1.9 ± 0.9	0.205
PIRO score	11.9 ± 4.6	11.8 ± 5.1	12.8 ± 4.3	0.045
MEDS score	8.8 ± 4.0	8.5 ± 4.1	9.1 ± 3.8	0.26
DNR status	89 (28)	71 (36)	18 (14)	<0.001
**ED Treatment**				
Fluids in ED (l)	1.9 ± 1.3	1.6 ± 1.1	2.2 ± 1.5	<0.001
Time to antibiotics (min)	115 ± 91	127 ± 95	94 ± 74	0.005
Number of goals attained	4.3 ± 1.4	3.9 ± 1.4	5.1 ± 1.0	<0.001
All goals attained	77 (24)	28 (14)	49 (39)	<0.001
**ICU consultation in ED**	171 (53)	46 (23)	125 (100)	<0.001
**Hospital length of stay** (days)				
Total	13.1 ± 15.0	10 ± 12	18 ± 19	<0.001
ICU	3.3 ± 8.9	0.5 ± 2.0	8 ± 13.1	<0.001
Ward	9.7 ± 10.4	9.7 ± 10.7	10.0 ± 10.1	0.789
**In-hospital mortality**	72 (22.3)	39 (20)	33 (26)	0.208

Data are presented as number (% of total) or mean (± SD). Abbreviations: COPD: chronic obstructive pulmonary disease; INR: International Normalized Ratio; ED: emergency department; ICU: intensive care unit; DNR: do not resuscitate; MAP: mean arterial pressure; PIRO: predisposition, infection, response, organ failure score; MEDS: Mortality in Emergency Department Sepsis score. †Immune compromised was defined as a patient having one or more of the following: currently on immune-suppressive medication, concurrent or recent chemotherapy, current hematological malignancy or medical history positive for acquired immune deficiency syndrome (AIDS) or human immunodeficiency virus (HIV).

### Outcome measures

Full compliance with the SSC resuscitation bundle was the main outcome measure of the present study. In addition, the effect of illness severity (as determined by the PIRO score) and disposition to ward or ICU on ED treatment was assessed.

### Data analysis

Descriptive continuous data are presented as mean ± SD or with 95% confidence intervals, unless indicated otherwise. Continuous variables were tested with Student *t*-tests. Categorical data were analyzed using *X*^2^ tests. To assess predictors of 100% compliance with the resuscitation bundle of the SSC, multivariable binary logistic regression was done with forward entry of variables that had *P* < 0.2 in univariate analysis. Regardless of the univariate analysis, clinical signs of organ failure, i.e., respiratory difficulty (defined as in PIRO score), initial systolic blood pressure <90 mmHg, altered mental status and febrile chills were entered in the multivariable analysis. Because both clinical and biochemical signs of organ failure are associated with mortality, the laboratory signs of organ failure (lactate > 4 mmol/l, urea >7.14 mmol/l, thrombocytopenia <150.10^12^/l) were also put in the model [17]. Liver dysfunction due to sepsis occurred in only three patients and was therefore not put in both models. The Hosmer-Lemeshow test was used as a measure of model calibration.

Sample size was based on the generally accepted rule of thumb of the number of events (in our study the number of patients in whom all goals of the SSC resuscitation bundle were attained) divided by ten. Because we wanted to put the aforementioned signs of organ failure in the model regardless of the univariate analysis, we needed ~70 events. In retrospect, merely 5 independent predictors of full compliance were identified and put in the final model, so ~50 events were needed, less than the 77 events in the present study.

To explore the relative impact of quality of ED treatment (as quantified by the number of SSC targets achieved), illness severity (as quantified by the PIRO score [17]), and disposition to the ward or ICU on mortality in our study cohort with relatively low mortality, we put these three variables in a binary logistic regression model with in-hospital mortality as an outcome measure in a similar way as described above. We expressed the effects of predictor variables on compliance and hospital mortality using odds ratios (ORs) including 95% confidence intervals (CIs).

Finally, because time to antibiotics is an important predictor of mortality [18-20], two-way analysis of variance was used to test if the time to antibiotics depends on illness severity and disposition. ICU admission was considered as a separate indicator of illness severity in addition to the initial PIRO score because it also incorporates the patient’s response to ED treatment. Non-responders to ED fluid resuscitation and patients with severe respiratory failure were expected to be admitted to the ICU.

All data were analyzed using PASW statistics18.0 (IBM, New York, USA) software.

## Results and discussion

### Results

**Figure 1:**
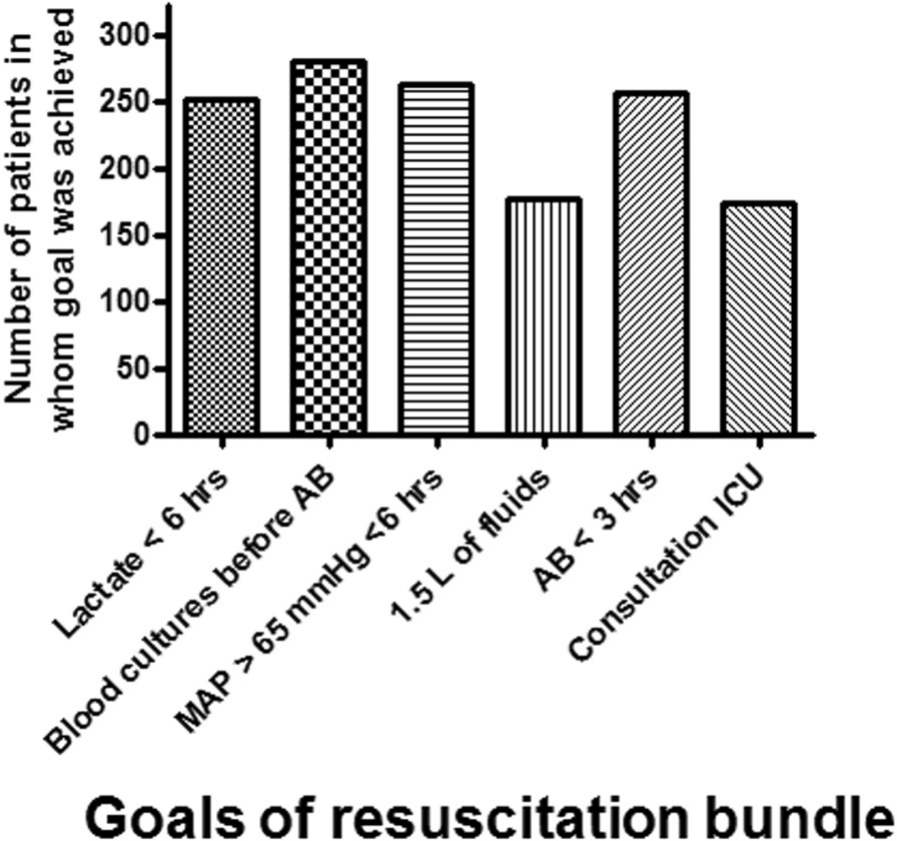
**Number of septic ED patients in whom a target of the resuscitation bundle had been achieved.** In the Netherlands, the targets of the resuscitation bundle requiring a central venous line are usually done in the ICU. ICU consultation was therefore considered as a goal to be attained in the ED.

In the study period ~212,527 patients visited both EDs. Three hundred twenty-three patients met the criteria for severe sepsis or septic shock. Table 1 shows patient characteristics. All goals were achieved in 24%. Figure 1 shows the number of patients in whom a specific goal was achieved.

### Factors associated with 100% compliance with the SSC guidelines

In Table 2, univariate and multivariate analyses are shown for predictors of 100% compliance. Illness severity *per se* was not an independent predictor of compliance, but clinically evident signs of organ failure, age and institution were. The time to availability of blood results was 63 (±39) min.

**Table 2:** Univariate and multivariate analysis of factors related to 100% compliance with the in the ED attainable goals of the resuscitation bundle of the “Surviving Sepsis Campaign”

**Variable**	**6 goals**	**<6 goals**	**P univariate**	**Corrected OR (95% CI) multivariate**
No. (%)	77 (24)	246 (76)		
**Patient-related factors**				
Age	62 ± 17	68 ± 17	0.005	0.98 (0.95–1.00)*
Male sex	48 (62)	135 (55)	0.29	
**Clinical signs**				
Respiratory difficulty (28)	49 (64)	114 (46)	0.006	3.38 (1.08–10.64)
Hypotension <90 mmHg (2)	37 (48)	84 (34)	0.043	2.37 (1.07–5.23)
Altered mental status (15)	42 (55)	76 (31)	<0.001	4.18 (1.92–9.09)
Febrile chills (30)	25 (32)	47 (19)	0.016	
**Laboratory findings**				
Lactate >4 (58)	26 (34)	74 (30)	0.41	
Urea >7.14 mmol/l (4)	50 (65)	184 (75)	0.102	
Thrombocytopenia (8)	23 (30)	44 (18)	0.036	
**Illness severity**				
Total PIRO score	12.9 ± 4.7	12.0 ± 4.9	0.149	
Total MEDS score	9.1 ± 3.8	8.7 ± 4.1	0.447	
**Institution-related factors**				
Academic (as opposed to urban)	46 (60)	58 (24)	<0.001	3.16 (1.44–6.94)^#^
Time of ED presentation:				
8.00 a.m.–23.30 p.m.	58 (75)	173 (70)		
23.30 p.m.–8.00 a.m.	19 (25)	73 (30)	0.648	
**Physician-related factors**				
ED physician involved (19)	28 (36)	52 (21)	0.01	
Admitting specialty				
Medical (the rest being surgical)	72 (94)	232 (94)	0.776	

Corrected OR (odds ratio, 95% confidence intervals) of >1 indicates that the factor is associated with higher odds of completing all goals. *OR per year increase of age. ^#^OR compared to urban set as 1. Clinically evident and laboratory signs of organ failure were defined as in the PIRO score [17]. Liver dysfunction was not shown since it occurred in only three cases because of sepsis. Dutch EDs are not fully staffed with ED physicians. Number of missing data is indicated between brackets in first column. If not mentioned no data were missing. Abbreviations: ED, emergency department; PIRO, predisposition, infection, response, organ failure score; MEDS, mortality in ED sepsis; model calibration: *P* = 0.867.

### The effect of illness severity and disposition on time to antibiotics

**Figure 2:**
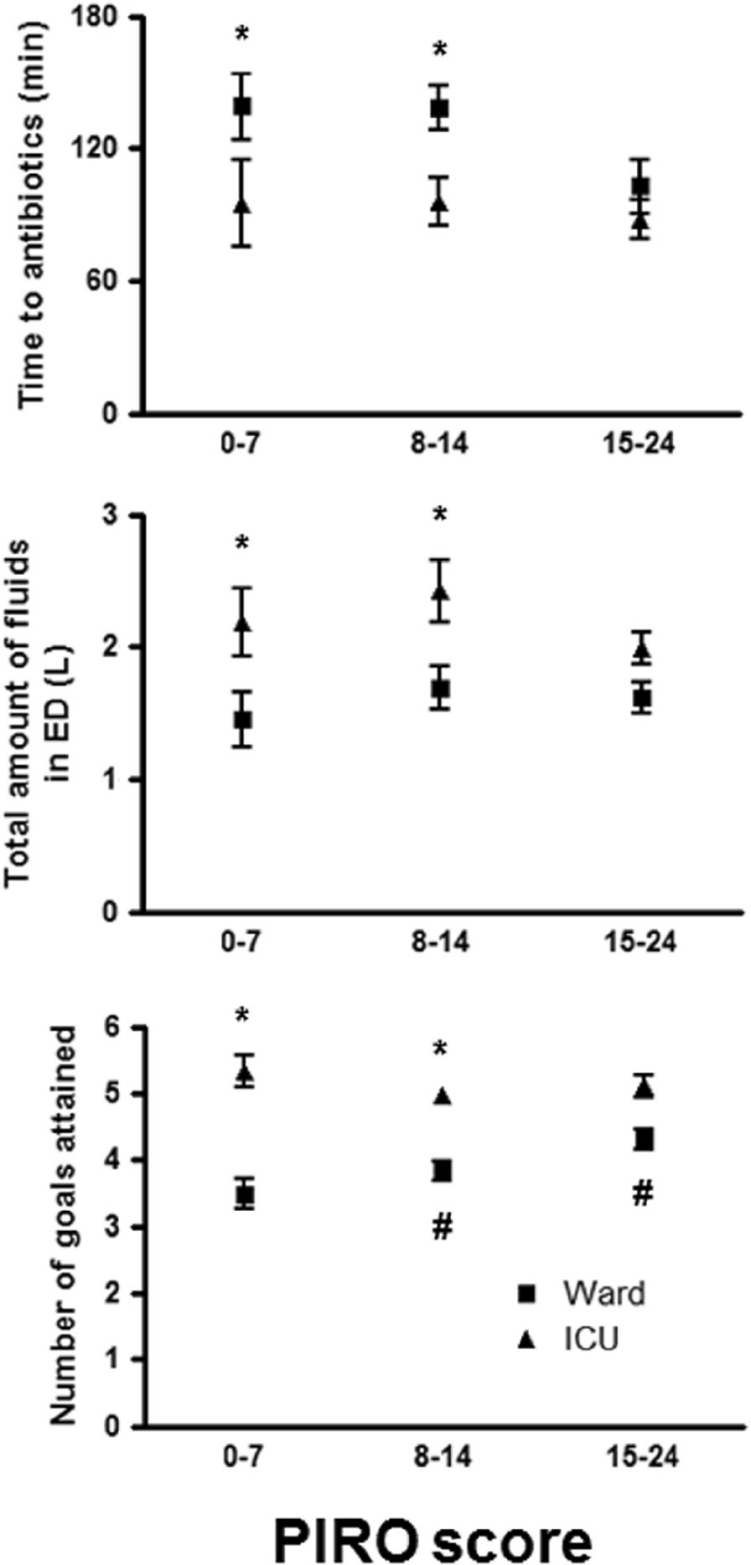
**Time to antibiotics, amount of fluids and number of goals achieved in the ED as a function of the PIRO score and stratified by disposition to the ward or ICU.** *Statistical difference between ward and ICU. #Statistically significant difference with PIRO score of previous category. Data are presented as mean ± standard error of the mean.

Early goal-directed therapy [21] cannot be completed in 198 of 323 patients admitted to a ward. In Figure 2 it is shown that, in ED patients admitted to the ICU, time to antibiotics is shorter, amount of administered fluids is larger and number of goals of the SSC resuscitation bundle achieved in the ED is higher in patients with PIRO score 1–14. Table 3 reveals that the percentage of clinically evident organ failure was higher in the ED patients admitted to the ICU compared to patients admitted to the ward.

**Table 3:** Univariate analysis of presence of clinical and laboratory signs of organ failure of septic ED patients admitted to the ward and ICU

**Variable**	**ICU**	**Ward**	***P***
No. (%)	125 (39)	198 (61)	
**Clinically evident signs of organ failure**			
Respiratory difficulty	70 (56)	89 (45)	0.034
Septic shock	36 (29)	27 (14)	<0.001
Altered mental status	54 (43)	59 (30)	0.003
Febrile chills	22 (18)	49 (25)	0.259
**Laboratory signs of organ failure**			
Lactate >4	42 (34)	56 (28)	1.0
Urea >7.14 mmol/l	85 (68)	143 (72)	0.895
Thrombocytopenia	28 (22)	38 (19)	0.335
**Total PIRO score**	12.8 ± 4.3	11.8 ± 5.1	0.054

Clinically evident and laboratory signs of organ failure were defined as in the PIRO score [17]. Liver dysfunction was not shown since it occurred in only three cases because of sepsis. Abbreviations: ED, emergency department; PIRO, predisposition, infection, response, organ failure score.

### The effect of illness severity, compliance and disposition on mortality

PIRO score was 11.5 ± 4.8 in survivors and 14.5 ± 4.1 in non-survivors (*P* < 0.001). Number of achieved goals was 4.3 ± 1.4 in survivors and 4.5 ± 1.2 in non-survivors (*P* = 0.379). Eighty-seven (35%) of the survivors were admitted to the ICU compared to 31 (43%) of the non-survivors (*P* = 0.18). The PIRO score was the only independent predictor of in-hospital mortality in our study cohort with relatively low mortality. The adjusted OR was 1.14 (1.07–1.21) per unit increase in the PIRO score.

#### Limitations

There are several limitations to our study. First, we did not register the age and years of experience of the attending physician, while in theory this could affect compliance. However, no studies investigating protocol adherence found an association between years of experience and compliance [22]. More importantly, in the Netherlands the ED is run by relatively young and inexperienced residents. The vast majority of doctors are still in training [23]. Consequently, there is no wide range of age and experience in Dutch EDs, and the assessment of the effect of age and years of experience is therefore difficult to explore.

Second, we could not assess all goals of the resuscitation bundle because the majority of patients were admitted to a normal ward and not to the ICU. In these patients, a central line was never inserted, and the full resuscitation bundle was consequently not provided. Therefore, we scored a point if the ICU was consulted. In addition, our study only supplied data with regard to compliance to the resuscitation bundle and not with the sepsis management bundle.

### Discussion

The present study has two main findings: First, the presence of clinically recognizable signs of organ failure is the most important factor associated with compliance to the resuscitation bundle of the SSC rather than illness severity *per se*, suggesting that lack of recognition of organ failure in ED patients with severe sepsis and septic shock plays a role in non-compliance to the SSC guidelines.

Secondly, septic ED patients with clinically evident signs of organ failure are more likely to be admitted to the ICU compared to patients with only biochemical signs of organ failure, which is associated with substantially shorter time to antibiotics, despite similar predicted mortality.

### Factors associated with 100% compliance with the SSC guidelines

The individual goals of the resuscitation bundle were achieved in 54 to 81% of the patients (Figure 1), but in only 24% all goals were attained despite an extensive Surviving Sepsis Campaign and the advice to start the resuscitation bundle as soon as organ failure was present as a sign of severe sepsis. Patients with clinical evidence of organ dysfunction received better patient care than patients with only laboratory evidence of organ failure, while their predicted mortality is similar since the two and three points in the PIRO score assigned for increased urea and lactate correspond with a similar increase in predicted mortality such as respiratory difficulty, shock and altered mental status (the latter in the MEDS score) [16,17]. Thus, despite the fact that clinical and laboratory variables give similar odds for mortality, they result in different compliance. Biochemical signs of organ failure were available within 63 min, well within the 3 h SSC target for time to antibiotics. Non-compliance was therefore not caused by waiting for availability of laboratory results. Instead, we hypothesize that clinically more ill-appearing patients are better recognized and are therefore treated better. An important implication of the present study is that EDs should incorporate specific tools for recognition of organ failure in the ED. The three screening questions of the SSC (suspected infection, SIRS criteria, organ failure, [1]) apparently trigger the attending physician in case of clinically recognizable signs of organ failure, but not when merely laboratory signs of organ failure are present, but these should lead to a similar sense of urgency. The use of information technology could increase the recognition of organ failure by coupling the clinical chemistry database with an electronic patient file that shows a warning when there is a biochemical sign of organ failure. This might be more effective than a simple screening list with signs of organ failure.

The ICU was more often involved in patient care in the LUMC, which might explain the higher odds for full compliance, consistent with the findings of Mikkelson and colleagues who showed that involvement of a severe sepsis service was associated with better compliance with early goal-directed therapy [22].

Finally, odds for full compliance decreases with a 2%/year increase in age, which is a worrisome finding, especially for older patients with a contraindication for ICU admission (i.e., DNR status) because in these patients optimal ED treatment might be the only treatment significantly improving prognosis. Our findings are consistent with the previously reported age-related differences in delivery of critical care [24,25], but the underlying etiology is unclear.

### The effect of illness severity and disposition on time to antibiotics

Timely administration of antibiotics and adequate fluid resuscitation are critical issues in septic ED patients [18-21]. Illness severity *per se*, as quantified by the PIRO score, was not associated with protocol adherence or time to antibiotics (Table and Figure 2). However, time to antibiotics was significantly shorter in patients admitted to the ICU, which might be partially explained by the observation that more ED patients with clinically evident signs of organ failure were admitted to the ICU (Table 3). In patients admitted to the ward, time to antibiotics was substantially longer, and this might affect mortality but also hospital length of stay [26]. The larger amount of fluids administered in ICU admitted patients might partially reflect poor responsiveness to fluid resuscitation, necessitating ICU admission, which is supported by the observation that more patients with septic shock are admitted to the ICU instead of the ward (Table 3).

### The effect of illness severity, compliance and disposition on mortality

Mortality was not associated with compliance, possibly because our study was underpowered to establish such a relationship. Treatment effect has been shown to depend on illness severity [27-29], which might also explain the absence of a correlation between compliance and mortality since in-hospital mortality was 22% in our study cohort compared to the 37 and 44% in the ICU population of previous studies investigating the effect of compliance to the SSC guidelines in patients admitted to the ICU [1,2].

## Conclusions

In summary, the presence of clinically recognizable signs of organ failure results in better compliance with the “Surviving Sepsis Campaign” compared to laboratory signs of organ failure. Septic ED patients with clinically evident signs of organ failure are more often admitted to the ICU as opposed to the ward, which is associated with shorter time to antibiotics. Recognition of severe sepsis is an important barrier to successful implementation of evidence-based quality improvement programs for ED sepsis patients.

## Consent

This study met the criteria for exemption from obtaining informed consent, because of the observational character of the study.

## Competing interests

The authors declare that they have no competing interests.

## Authors’ contributions

BDG invented the study idea, designed the study, collected data, contributed to the analyses and edited the manuscript. BDG takes full responsibility for the study as a whole. DK collected data, did the analyses and wrote the manuscript. MHS edited the manuscript. AV and ERJTD collected data. All authors read and approved the final manuscript.
